# Understanding how emotional intelligence shapes teachers’ reflective and creative teaching: the moderating role of teacher autonomy

**DOI:** 10.3389/fpsyg.2026.1746923

**Published:** 2026-02-11

**Authors:** Şenol Orakci, Osman Aktan, Hüseyin Çevik

**Affiliations:** 1 Faculty of Education, Curriculum and Instruction, Aksaray University, Aksaray, Türkiye; 2 Faculty of Education, Special Education, Düzce University, Düzce, Türkiye; 3 Faculty of Education, Curriculum and Instruction, Zonguldak Bülent Ecevit University, Zonguldak, Türkiye

**Keywords:** creative teaching, emotional intelligence, reflective teaching, teacher autonomy, teachers

## Abstract

**Introduction:**

Emotional intelligence is a key predictor of teachers’ instructional behaviors, yet its role in shaping reflective and creative teaching remains insufficiently understood. Little evidence explains how emotional intelligence promotes creative teaching through reflective practice or how teacher autonomy conditions this process. This study examines a moderated mediation model linking emotional intelligence, reflective teaching, creative teaching, and teacher autonomy.

**Methods:**

Using a cross-sectional survey design, data were collected from 690 teachers in 78 schools across 24 Turkish cities. Validated scales measured emotional intelligence, reflective teaching, creative teaching, and autonomy. Multilevel structural equation modeling with Bayesian estimation tested indirect and moderated effects.

**Results:**

Emotional intelligence showed a significant indirect effect on creative teaching through reflective teaching. Teacher autonomy strengthened this indirect pathway, amplifying the positive influence of emotional intelligence on reflection and, in turn, creative instruction.

**Discussion:**

Findings highlight the central roles of emotional intelligence, reflective practice, and autonomy in fostering creative teaching. Enhancing teachers’ emotional skills, reflective capacities, and autonomy may support more innovative instructional practices and improved learning outcomes.

## 1 Introduction

Teachers’ emotional intelligence (EI)—their ability to perceive, understand, regulate, and utilize emotions—has been increasingly recognized as a key determinant of effective teaching practice (e.g., Jiang and Tong, 2025; Su et al., 2022). Evidence shows that emotionally intelligent teachers are more adept at fostering student engagement, addressing classroom challenges, and implementing innovative pedagogies (Aponte et al., 2025; Jiang and Tong, 2025). However, the mechanisms and contextual factors that explain how EI translates into creative teaching (CT) remain underexplored. This study addresses that gap by testing a moderated mediation model in which reflective teaching (RT) mediates the relationship between EI and CT, while teacher autonomy (TA) moderates this indirect link. RT—defined as systematic self-examination of instructional beliefs, decisions, and experiences—serves as the mechanism through which EI fosters pedagogical creativity. Emotionally intelligent teachers are more likely to reflect on and refine their practices, thereby developing innovative instructional strategies that enhance learning outcomes (e.g., Abdulkader and Al Naggar, 2024; Jiang and Tong, 2025). TA-the professional discretion to make instructional and organizational decisions—functions as a boundary condition for this process. Greater autonomy enables teachers to transform reflective insights into creative practices, whereas restricted autonomy may constrain such enactment. Prior research has linked autonomy to teachers’ professional commitment and innovative capacity (e.g., Chen, 2024; Ertürk, 2023). Despite these theoretical connections, empirical tests of this conditional process remain scarce. Clarifying how and when EI promotes CT can inform emotion-based professional development and policy initiatives that foster reflective practice and enhance TA. Given that EI is developable and positively influences instructional innovation (e.g., Jiang and Tong, 2025; Su et al., 2022), examining this moderated mediation model provides both theoretical and practical insight.

Accordingly, this study empirically tests a multilevel model positioning RT as the mediator linking EI to CT and TA as the moderator of this pathway. The findings are expected to illuminate the mechanisms and institutional conditions under which EI contributes to creative pedagogy, offering actionable implications for teacher education, leadership, and policy aimed at promoting more innovative and engaging classrooms.

### 1.1 The Turkish educational context

Different cultures have varying expectations and perceptions regarding education. The Turkish educational system, with its unique cultural and societal elements, necessitates a specific exploration of the relationship between EI, CT, RT, and TA. Türkiye has been undergoing educational reforms and changes in policies that emphasize certain teaching methodologies. Investigating the relationships between EI, CT, RT, and TA could be crucial in aligning teacher practices with the evolving educational landscape.

Understanding how EI influences creativity and RT may be essential for designing effective professional development programs for teachers in Türkiye. Tailoring training to the local context can enhance its relevance and impact. The Turkish education system has specific goals for student outcomes, and understanding how EI and teaching styles contribute to these outcomes can justify the study. For instance, there has been an emphasis on fostering creative and critical thinking skills in the Turkish education system (Orakci and Durnali, 2023; Taneri, 2012). Within this context, the study can explore how reflective and CT practices contribute to this goal. On the other hand, socioeconomic factors in Türkiye influence the dynamics of the classroom. Investigating the relationship between EI, reflection and CT can also provide insights into how teachers navigate these factors to create effective learning environments. Examining the connection between EI and autonomy can also shed light on the well-being of teachers in Türkiye.

Finally, the literature on the relationship between EI, CT, RT, and TA is rather limited in the context of Türkiye. Bridging this gap by conducting the present study can contribute valuable insights to both local and global educational research.

In the light of the information given above, the concepts of “creative teaching” (CT), “reflective teaching” (RT), “emotional intelligence” (EI) and “teachers’ autonomy” (TA) are thought to form the basis of qualified teaching. In the present study, we investigated a moderated mediation model of EI’s effects on CT, involving RT as mediator and TA as moderator. This study sought to answer the following questions:

1. What are the direct effects of EI on RT, and the direct and indirect effects of EI on CT?
2. Does RT mediate the effects of EI on CT?
3. Does the level of TA moderate the effects of EI on CT through RT?

**Figure 1:**
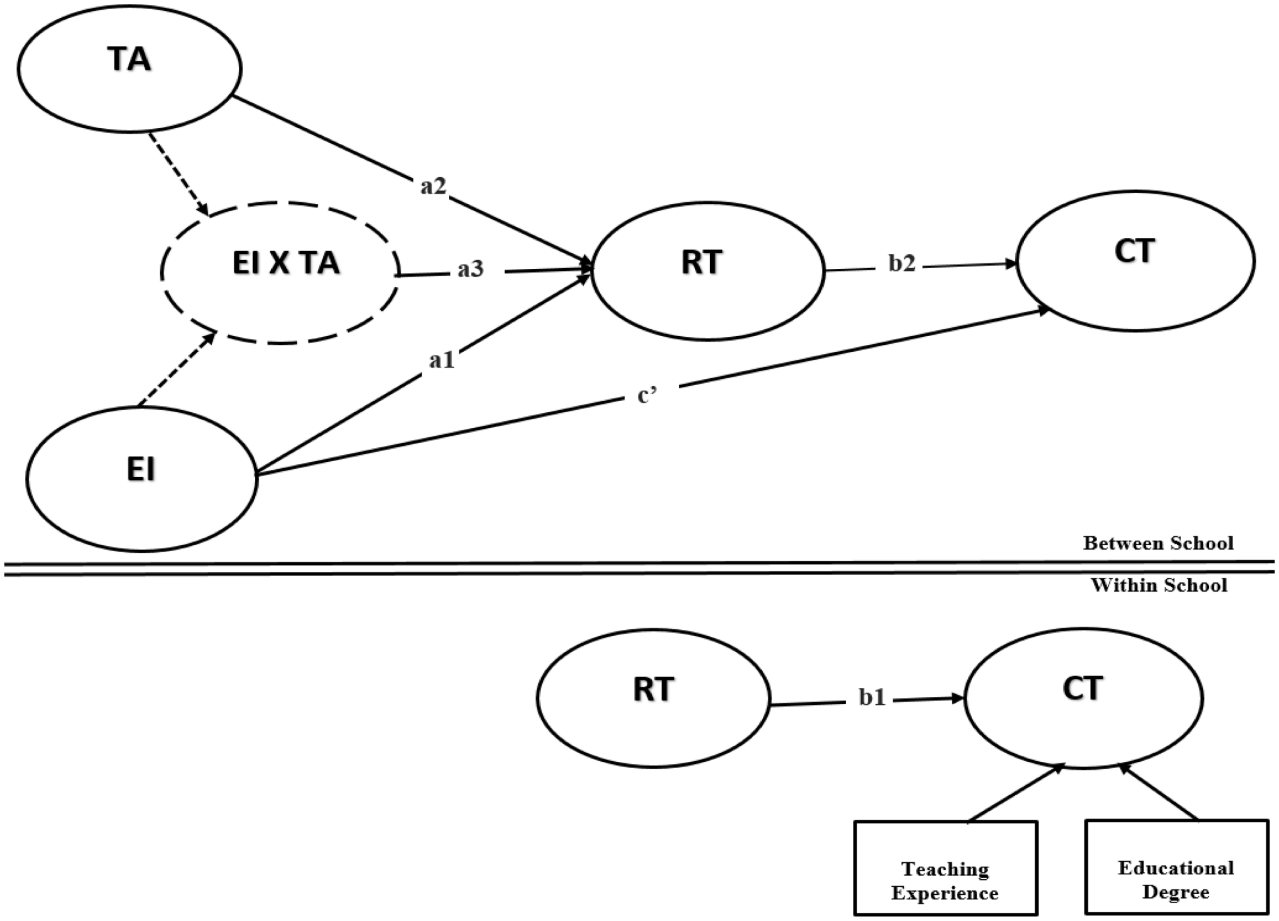
Hypothetical model.

To extend previous studies by investigating the moderation effect of TA on the association between EI and CT, with the mediating effect of RT, our conceptual framework is presented in Figure 1.

Our conceptual model in Figure 1 presents the following hypotheses below:

*H1*: RT will be positively related to CT.

*H2*: EI will be positively related to RT.

*H3*: EI will be positively related to CT.

*H4*: The influence of EI on CT will be realized through an indirect pathway, possibly involving the mediating role of RT.

*H5*: The relationship between EI and RT will be moderated by TA, such that the relationship will be stronger when teachers’ perception of TA is stronger.

*H6*: EI will be related to CT via conditional indirect effects, such that its association with CT will be moderated by TA and mediated by RT.

### 1.2 Creative teaching

CT represents a dynamic and contextually responsive approach in which educators design and facilitate learning experiences that extend beyond routine practice, inviting student imagination, inquiry, and flexible problem-solving (Wei and Chuang, 2024). In this paradigm, teachers draw on deep subject knowledge and pedagogical reflection to craft activities that are not just novel but meaningfully connected to learners’ interests and real-world problems, thereby stimulating engagement and deeper understanding (Muñoz-Salinas et al., 2025). Importantly, research indicates that when school environments provide strong support—such as autonomy in instructional design, collaborative professional learning, and access to resources—teachers are far more likely to sustain creative practices in the classroom. Conversely, structural constraints such as rigid curricula, high stakes accountability, and limited time undermine such efforts, pointing to the critical role of institutional and policy contexts in unlocking teachers’ creative potential (OECD, 2025). CT hinges upon the teacher’s ability to translate reflective insight into adaptive instructional practice. As noted by Creative Teaching in the Context of Educational Reform: Insights from the Perspective of In service Teachers (Wei and Chuang, 2024), in service teachers conceptualize CT less as the invention of entirely new pedagogical paradigms and more as the strategic adaptation of instructional methods to changing contexts, integration of multidisciplinary content, and commitment to ongoing refinement of practice. This recognition underscores the role of teacher metacognition: CT flourishes where educators actively monitor and revise their planning, enactment, and student responses, thereby making originality and relevance a continuous iterative process.

Moreover, the organisational context and structural affordances play a pivotal role in enabling or constraining CT. Research such as TA, Creative Self efficacy, and Innovative Behavior: Perspectives from Chinese University EFL Teachers shows that higher levels of perceived TA are positively associated with creative self efficacy, which in turn bolsters teachers’ innovative classroom behavior. When teachers believe they have meaningful decision making authority over instructional planning, assessment strategies, and classroom environment, they are more likely to engage in divergent, exploratory teaching designs rather than purely routine implementations (Chen, 2024; Martín-Ezpeleta et al., 2024). Conversely, tightly scripted curricula, high stakes accountability systems, and minimal professional discretion have been found to suppress creative initiatives, signaling that cultivating CT demands not only individual teacher capacity but collectively structured professional ecosystems.

### 1.3 Relationship between reflective teaching and creative teaching

RT refers to a structured process through which educators systematically examine their instructional decisions, student responses, and classroom interactions in order to improve professional practice and student learning outcomes. Recent research emphasizes that reflective approaches not only enhance teachers’ ability to adapt and innovate but also strengthen their self-regulation and pedagogical responsiveness (Ghorbani Moghaddam, 2023; Velásquez et al., 2023). In teacher education, fostering reflection via tools such as teaching journals, peer discussion, and micro-teaching has been shown to bolster novice teachers’ confidence and readiness for real-world classrooms (Toker, 2023). Overall, RT is increasingly recognized as a key component of teachers’ ongoing professional growth in dynamic educational contexts.

The interplay between CT and RT is increasingly recognized as central to dynamic instructional practice. RT enables educators to analyse and adapt their instructional decisions, attend to student responses, and iteratively refine their methods (Khasawneh, 2024). This continuous self-examination creates fertile ground for CT, as teachers who engage in systematic reflection are better positioned to identify opportunities for change, experiment with novel approaches, and respond flexibly to classroom dynamics. In essence, reflection acts as a precursor to creativity, equipping teachers with insights into when and how to depart from routine pedagogy. Furthermore, CT extends and actualises reflective insight by transforming thoughtful critique into instructional innovation. In a study of in-service teachers, it was found that CT was not necessarily about inventing entirely new methods, but about adaptively integrating multidisciplinary content, using unconventional strategies, and continually modifying practice in light of reflective processes (Wei and Chuang, 2024). Thus, the reflective teacher becomes the creative teacher when reflection informs experimentation and when innovation becomes grounded in a cycle of review, planning, action, and re-reflection. The relationship is thus reciprocal: creativity invites further reflection, and reflection supports sustainable innovation.

Emerging evidence indicates that RT not only strengthens teachers’ self awareness and instructional adaptability, but also lays the foundation for creative pedagogical practices. For instance, in a teacher training context, participants who engaged in systematic reflection exhibited greater creative thinking competences (Soto-González et al., 2023). Moreover, professional development focused on reflective practices improved teacher student interaction quality, a pre condition for flexible, inventive instructional designs (Khasawneh, 2024). A recent comprehensive review of teacher creativity further identifies reflective practice as a key enabling factor of CT (Martín-Ezpeleta et al., 2024; Nga et al., 2025). In this context, creativity is described as one of the high-level cognitive abilities that is strongly related to reflective thinking. Considering this theoretical foundation and the acquired evidence, we hypothesized that “RT will be positively related to CT (Hypothesis 1).”

### 1.4 Relationship between emotional intelligence and reflective teaching

EI denotes the capacity to perceive, understand, manage, and use emotions effectively in oneself and in relations with others; within education, teachers’ EI supports adaptive classroom management, constructive teacher–student interactions, and professional resilience (Aldrup et al., 2024). Recent empirical studies show that higher teacher EI is associated with greater psychological well-being and organizational commitment, and that EI relates to practical outcomes (e.g., reflective practice and innovative behaviors) via proximal mechanisms such as emotion regulation and empowerment (Fuentes-Vilugrón et al., 2024; Li et al., 2024). Consequently, EI is best understood as a foundational, malleable resource in teacher development that interacts with contextual supports (e.g., autonomy, leadership) to shape reflective and creative instructional practices. In fact, teachers’ EI—the ability to perceive, understand, regulate, and use emotions in oneself and in relationships—is increasingly recognized as foundational for effective RT. For example, a study of Iranian EFL teachers found significant positive correlations between all five EI domains (self-awareness, self-management, social awareness/empathy, relationship management, and motivation) and teachers’ RT ability; among these, self-awareness and motivation were the strongest predictors of reflectivity (Ferdosipour and Salimi, 2023). This suggests that emotionally intelligent teachers are more likely to engage in the intentional planning, monitoring, and evaluation cycles characteristic of RT.

The relationship between EI and RT can be understood as a dynamic interplay: high EI provides the intrapersonal and interpersonal scaffolding necessary for authentic reflection, while RT offers the cognitive-metacognitive processes by which emotionally aware teachers translate their emotional insights into instructional refinement. This dynamic is particularly relevant in challenging or shifting educational contexts (e.g., online teaching, pandemic-related transitions) where teachers’ emotional responses influence whether they engage in critical self-reflection and subsequently redesign their teaching practice (Chiu, 2025). Dogham et al. (2025) revealed a strong positive correlation between EI (especially emotionality, self-control, sociability) and reflective thinking among students. Although in the nursing domain, this suggests a parallel mechanism—higher EI supports more effective reflective processes. Cross-cultural research among teacher education students by Kyriazopoulou et al. (2025) found that EI supports metacognitive and affective dispositions. These dispositions are also central to RT, suggesting an indirect pathway from EI to reflection. Considering the theoretical foundation and relevant evidence, we posit that “EI will be positively related to RT (Hypothesis 2).”

### 1.5 Relationship between emotional intelligence and creativity

EI, defined as the ability to perceive, understand, manage, and use emotions in oneself and others, has increasingly been recognized as a facilitator of creative thinking and behaviors. A meta-analysis of 75 studies found a moderate positive correlation (r ≈ 0.32) between EI and creativity, suggesting that individuals with higher levels of EI tend to exhibit greater creative potential. Emotionally intelligent individuals, for instance, are more adept at regulating negative affect and sustaining positive affect, both of which contribute to cognitive flexibility and divergent thinking—key components of creativity (Xu et al., 2019). More specifically, trait EI has been shown to predict creative self-efficacy in children and adolescents, which in turn supports creative output (Cheng et al., 2024).

Empirical findings indicate that EI can influence teachers’ creative instructional behaviors through intermediary variables linked to self-regulation, self-efficacy, and metacognitive reflection. Longitudinal studies with pre-service and in-service teachers show that higher EI predicts enhanced reflective thinking, which subsequently promotes innovative classroom practices (Jiang and Tong, 2025; Shi et al., 2025). This pattern supports a model in which EI does not merely correlate with creativity in isolation but operates via reflective processes that translate emotional awareness and regulation into concrete pedagogical actions. Finally, conceptual models and chain-mediation analyses further reinforce the mediating role of RT. For instance, research in teacher education demonstrates sequential mediation, where EI influences psychological empowerment and reflective engagement, which then fosters creative or innovative teaching behaviors (Jiang and Tong, 2025). Collectively, these findings provide both theoretical and practical support for the hypothesis that RT mediates the relationship between teachers’ EI and their CT outcomes. Taking into account the underlying theories and the body of available evidence, we hypothesize that “EI will be positively related to CT (Hypothesis 3).”

Based on “Hypotheses 1–3 that RT will be positively correlated with CT and that EI will be positively and directly related to RT and CT,” our conceptual model presents a fourth hypothesis: “the effects of EI on CT will be accrued indirectly, with the possible mediating function of RT (Hypothesis 4).”

### 1.6 Relationships among emotional intelligence, creative teaching, reflective teaching, and teacher autonomy

Educators’ EI their ability to perceive, regulate, and harness emotions in themselves and in interactions with students—has emerged as a foundational component supporting RT and subsequently CT. In particular, emotionally intelligent teachers are more likely to engage in reflective practices: they monitor their emotional responses to classroom events, consider how these responses influence instructional decisions, and refine their practice accordingly. This reflective cycle provides the basis for innovation in pedagogy, connecting EI with higher-order instructional change (Jiang and Tong, 2025).

RT, in turn, acts as a bridge linking TA to CT. When teachers have professional discretion over curriculum adaptation, instructional methods, and assessment design, their reflective insights are more likely to translate into meaningful innovation (Sun and Yuan, 2024). In contexts with higher TA, the reflection-innovation connection is strengthened: teachers not only think critically about their practice but also enact novel, student-centred strategies. This suggests that autonomy moderates the pathway from RT to CT behaviors.

Finally, the synergistic interplay among these constructs implies that CT is most likely realized when a teacher combines EI, reflective practice, and professional autonomy. Empirical work indicates that TA supports the translation of both EI and RT into creative instructional behaviors (Pashazadeh and Alavinia, 2019). In such models, EI supplies the meta-emotional and regulatory resources, RT provides the cognitive-metacognitive mechanism, and autonomy offers the structural freedom needed to implement creative pedagogy. Together, these elements form a systemic network supporting the development of CT in contemporary educational settings.

### 1.7 The moderating role of teachers’ autonomy

TA, which is another concept analysed in the study, corresponds to the ability of teachers to reveal their independent existence with their own voices in decision-making processes in educational environments. Our review of the literature reveals controversy over the definition of TA and there are different points of view defining TA as the skill/capacity a teacher has and the specificity given to him. In this direction, TA is the ability to cultivate professional attitudes and skills through collaboration (Smith, 2000); the ability of teachers to freely structure their teaching (Javadi, 2014); the ability of teachers to provide self-directed instruction (Little, 1995), and decision-making skills (Pearson, 1995). Likewise, Shaw (2002) argues that the level of autonomy a teacher feels rests on one’s own skill and capacity. From a theoretical standpoint, autonomy provides the structural freedom necessary for reflection and innovation: when teachers feel empowered to make pedagogical decisions, the relationship between EI and RT is more likely to translate into meaningful instructional change. Empirical evidence supports this: in a sample of Chinese university EFL teachers, TA positively predicted creative self-efficacy and innovative behavior, suggesting autonomy enhances the translation of cognitive and emotional resources into practice (Chen, 2024). Thus, autonomy can strengthen the pathway from EI → RT by enabling teachers to act upon reflective insights.

The moderating function of TA in the EI-RT relationship is logically grounded in self-determination and professional agency theories. Teachers with high EI possess the intrapersonal and interpersonal competencies (e.g., emotion regulation, empathy, self-motivation) necessary to reflect deeply on their practice. However, without sufficient autonomy, such reflective insights may remain abstract or constrained by externally mandated curricula and tight accountability regimes. Studies examining TA in lesson planning underscore this point: Narayanan et al. (2023) found that perceived autonomy over instructional design moderated teachers’ capacity to engage creatively when reflection alone was not enough to effect change. In this context, autonomy serves as a boundary condition: the relationship between EI and RT is stronger when autonomy is high and weaker when it is low.

In summary, TA serves as a pivotal moderator in integrated models of CT. In a full moderated-mediation framework, EI influences CT via RT, but the strength of this indirect pathway depends on TA. When teachers perceive high autonomy, their EI is more likely to lead to deep reflection, which in turn fosters creative instructional practices. Conversely, low autonomy may stifle the translation of EI into RT and subsequently into CT. As such, educational interventions aiming to enhance CT would benefit from not only developing EI and reflective practices, but also from fostering greater TA to enable those competencies to be enacted in the classroom. Considering the results of prior research findings, we hypothesized that “the relationship between EI and RT will be moderated by TA, such that the relationship will be stronger when teachers’ perception of TA is stronger (Hypothesis 5).” Taking into account “Hypotheses 1–5 asserting that RT will be related to CT and that the correlation between EI and RT is moderated by TA,” we pose our final hypothesis as “EI will be related to CT via conditional indirect effects, such that its association with CT will be moderated by TA and mediated by RT (Hypothesis 6).”

## 2 Research method

We used a cross-sectional survey design in the present study. The sample, data collection procedure, psychometric features of the measures, control variables and analytical strategy were described in the following section.

### 2.1 Sample

The study was conducted in early 2023. Based on a convenience sampling method, the voluntary participants were 690 teachers from 78 schools across 24 cities in Türkiye. 385 of the participants were female and 305 were male. The age range of the participants was 25–62, with a mean of 32.5. Of the teachers, 92 (13.3%) are “Turkish Language and Literature” teachers, 85 (12.3%) “English” teachers, 82 (11.2%) “Turkish” teachers, 77 (11.1%) “Science” teachers, 55 (8%) “Geography” teachers, 54 (7.8%) “History” teachers, 53 (7.7%) “Religious Culture and Moral Knowledge” teachers, 44 (6.4%) “Mathematics” teachers, 31 (4.5%) “Social Science” teachers, 29 (4.2%) “Counseling and Guidance” teachers, 27 (4%) “Special Education” teachers, 21 (3%) “Primary School” teachers, 21 (3%) “Music” teachers, and 19 (2.7%) “P. E” teachers. Of the teachers, 518 (75%) held a bachelor’s degree, 159 (23%) held a master’s degree, and 13 (2%) held a doctoral degree.

### 2.2 Data collection tools

Firstly, ethical approval for our study was obtained from the Human Research Ethics Committee of Aksaray University, where one of the researchers is employed (Protocol No: 9, Date: February 7, 2024).

The “*Teacher Autonomy Scale*” (TAS) was developed and validated by Ulaş and Aksu (2015). It is a three-dimensional Likert-type scale with 18 items (e.g., “I feel autonomous to select the topics for the annual/daily plans.” “I feel autonomous to specify the aims and objectives for my instruction.”) and 5-points (1 = not at all 5 = extremely). “Cronbach’s alpha coefficient” overall was 0.81; 0.82 for the “Autonomy in Instructional Planning and Implementation” dimension; 0.74 for the “Autonomy in Professional Development” dimension; and 0.84 for “Autonomy in Determining the Framework of the Curriculum” dimension for this study.

The “*Emotional Intelligence Scale*” (EIS) developed and validated by Austin et al. (2004) was adapted into Turkish by Tatar et al. (2017). The scale with three constructs consists of 41 items (e.g., “My mood has little effect on how I deal with problems.,” and “I quite often misread what is going on in social situations.”). “Cronbach’s alpha coefficient” overall was 0.84; 0.83 for the “Optimism/Mood Regulation” dimension; 0.79 for “Utilisation of Emotions” and 0.81 for the “Appraisal of Emotions” dimension for this study.

“*Teaching for Creativity Scale*” (TFCS) developed and validated by Rubenstein et al. (2013) was adapted into Turkish by Yalçın and Çiçekler (2021). It is a four-dimensional Likert-type scale with 36 items (e.g., “I am capable of helping students to become more flexible in their thinking.” “My school’s priorities do not include teaching students to think creatively.”). “Cronbach’s alpha coefficient” overall was 0.85; 0.82 for the “Teacher Self-Efficacy” dimension; 0.74 for the “Environmental Encouragement” dimension; 0.84 for the “Societal Value” dimension, and 0.85 for the “Student Potential” dimension for this study.

The “*Teacher Reflection Scale*” (TRS) was developed and validated by Akbari et al. (2010) and adapted into Turkish by Korumaz (2012). It is a five-dimensional Likert-type scale with 29 items (e.g., “I talk about my classroom experiences with my colleagues and seek their advice/feedback.,” “I read books/articles related to effective teaching to improve my classroom performance.”) and 5-points (1 = Always to 5 = Never). The “Cronbach’s alpha coefficient” overall was 0.81; 0.80 for the “Practical” dimension”; 0.79 for “Learner (affective)” dimension; 0.80 for “Meta-cognitive” dimension; and 0.78 for “Critical” dimension for this study. The results of preliminary analyses for the fit indices of four scales are given in Table 1.

**Table 1:** The fit indices of the scales.

Scales	The fit indices						
	*X^2^/df*	*RMSEA*	*CFI*	*GFI*	*IFI*	*AGFI*	*SRMR*
Teacher Autonomy Support	3.12	0.07	0.90	0.88	0.89	0.84	0.06
Emotional Intelligence Scale	4.80	0.05	0.88	0.89	0.89	0.87	0.05
Teaching for Creativity Scale	2.98	0.07	0.88	0.87	0.86	0.88	0.05
Teacher Reflection Scale	3.62	0.07	0.91	0.95	0.91	0.92	0.06

On the whole, the fit indices derived from confirmatory factor analyses (CFA) of the four scales pointed out acceptable fit (Cole, 1987; Gliem and Gliem, 2003; Kline, 2011; Tabachnick and Fidell, 2013).

In our study, education degree and teaching experience were also accepted as control variables in our analyses because the previous studies (e.g., Setiawan, 2017) proved that education degree and teaching experience influence teachers’ creatıve thinking.

### 2.3 Analytical strategy

We used Multilevel Structural Equation Modelling (MSEM) with Bayesian estimation in “Mplus 8.7” to test our hypotheses with regard to the teacher and school levels (Muthén and Muthén, 1998–2017). Prior to performing the analysis, we used the “Mahalanobis Distance” to check for “multivariate normality” and to omit outliers. After that, we computed “mean,” “standard deviation,” and “zero-order correlations.” Additionally, with the aim of investigating aggregation for the school-level variables (EI and TA), we used the “within-group agreement (Rwg)” and the “intra-class correlations coefficient ICC (1)” and “ICC (2)” (Raykov et al., 2016). As a result, all of our variables had “Rwg” values over. 70 (Lüdtke and Robitzsch, 2008), and the variables had a strong within-group agreement. The “intra-class correlation coefficient (ICC) (1)” for each measure was also higher than.05, showing that the combination of teachers’ scores into the school level is appropriate.

The “ICC (2)” for each measure varied from 0.63 to 0.87 in the context of the assessment of the reliability of the school-level means, indicating good indices (Shrout and Fleiss, 1979). In our model, we used “confidence interval (CI)” coverage to evaluate the significance and strength of the impacts of the variables. We found that if the “CI” does not include zero, 95% of the “CI” for each latent variable’s effects are significant (Preacher and Hayes, 2008). For the fit indices in the “Bayesian method,” the “Potential Scale Reduction (PSR)” values should be around 1 (iteration = 10,000). By using the “Wald Chi-square test,” we also analyzed “Chi-square/degree of freedom” (2/df), “Log-Likelihood (LL),” “Akaike Information Criterion (AIC),” “Bayesian Information Criterion (BIC),” and “Standardized Bayesian Information Criterion (SABIC)” regarding the standards of parameters (Nylund et al., 2007). To assess the “moderating effect” of “TA,” we lastly computed the “conditioned indirect effect” of “EI” on “CT” through “RT” at “low” (2, 1 SD), “medium (mean),” and “high” (+ 1, + 2 SD) “moderator levels” (Table 2)

**Table 2:** Interrater agreement and interrater reliability of variables.

Index	Emotional intelligence	Teacher autonomy	Reflective teaching	Creative teaching
ICC1	0.387	0.289	0.347	0.156
ICC2	0.875	0.821	0.845	0.634
^R^wg	0.959	0.965	0.978	0.837

ICC, intraclass correlation coefficient; Rwg, within-group agreement.

### 2.4 Initial analysis of the variables

Based on the computations of all variables, we found that the mean values for our variables were higher than average: EI (“M” = 4.17, “SD” = 0.74), TA (“M” = 4.79, “SD” = 0.75), RT (“M” = 5.63, “SD” = 0.76) and CT (“M” = 4.13, “SD” = 0.57). We revealed that the “skewness” and “kurtosis” values for all variables ranged between −2 and + 2 (George and Mallery, 2010), proving that the normality assumption was true. We found a positive correlation between EI and RT (*r* = 0.649, *p* < 0.001), EI and CT (*r* = 0.537, *p* < 0.001) as well as RT and CT (*r* = 0.569, *p* < 0.001) at the school level. Additionally, we found a positive correlation between TA and RT (*r* = 0.562, *p* < 0.001) as well as TA and CT (*r* = 0.609, *p* < 0.001). After that, we used “MSEM Bayesian computation” to examine the intercorrelation between our variables. The “tolerance index (TI > 0.20)” and “variation inflation factor (VIF 5)” were used in “multivariate statistical analysis” to determine whether a “multicollinearity” issue existed. As a result, the range of “VIF” values was 1.11 to 3.82, and TI values ranged from 0.28 to 0.92, showing no evidence of a “multicollinearity” issue. In light of this, we described the direct and indirect effects among variables (Hypotheses 1–4), coupled with the moderation effect of TA in the association between EI and RT (Hypothesis 5), and the moderation effect of TA on the association between EI and TIP through CT (Hypothesis 6) (Table 3).

**Table 3:** “Descriptive statistics and correlations” between the variables.

Variable		M	SD	1	2	3	4	5	6	7	8
Teacher level (*n* = 690)											
1.	RT	–	–	–							
2.	CT	–	–	0.482**	–						
3.	Teac. Exp.	–	–	0.047	0.052	–					
4.	Ed. level	11.63	7.84	0.082*	−0.007	−0.139**	–				
School level (*n* = 78)											
5.	RT	5.63	0.76					–			
6.	CT	4.13	0.57					569**			
7.	TA	4.79	0.75					0.562**	609**	–	
8.	EI	4.17	0.74					0.649**	0.537**	489**	–
Skewness		–	–	−0.547	−0.685	−0.545	0.579	−0.575	−0.663	0.939	0.736
Kurtosis		–	–	0.328	1.325	−1.850	−0.287	0.329	1.307	1.321	0.923

M, means, SD, standard deviation; ICC, intraclass correlation; RT, reflective teaching; CT, creative teaching; TA, teacher autonomy; EI, emotional intelligence; Teac. Exp, teaching experience; Ed. level, education level.

**p < 0.01, *p < 0.001.

### 2.5 Model testing

Before adding additional variables to the analysis in Model 1, we first tested our first hypothesis. Next, we analysed the “Bayesian fit indices” of our final model. We noticed that the “PSR” value was close to 1 (“PSR” = 1.297). We also performed the “Wald Chi-square test” for the standards of the parameters, and the results showed that our data produced a satisfactory fit (χ2/df = 4.825, *p* > 0.01). For the first model, the information criteria were determined to be (9762.870) for “LL,” (18967.802) for “BIC,” (19289.929) for “AIC,” and (19845.690) for “SABIC.” As more variables were involved in the final model, the values of “LL” (8790.489), “BIC” (19389.485), “AIC” (18702.563), and “SABIC” (18789.652) declined. This indicates a better fit for our final model (Nylund et al., 2007). In order to interpret the findings more meaningfully, effect size (ES) (Cohen’s f2) was computed. Table 4 below shows that the initial model had a good fit with the data (TLI = 0.967, CFI = 0.973 and RMSEA = 0.058), which means that our measurements of the latent model variables were acceptable. The revised model also displayed a good fit with the data, as considered by the proposed criteria (TLI = 0.967, CFI = 0.980 and RMSEA = 0.050). As a result, the proposed research model as the final model was verified due to its more conciseness.

**Table 4:** The goodness-of-fit comparison between the initial model and the revised model.

Model	*X^2^/df*	*RMSEA*	*TLI*	*CFI*	*GFI*	*IFI*	*AGFI*	*SRMR*
Initial model	4.825	0.058	0.967	0.973	0.88	0.89	0.84	0.06
Revised model	4.785	0.050	0.967	0.980	0.89	0.89	0.87	0.05
Criteria (Byrne, 2012)	<5	< 0.70	> 0.90	> 0.90	> 0.90	0.86	≥0.80	< 0.80

**Figure 2:**
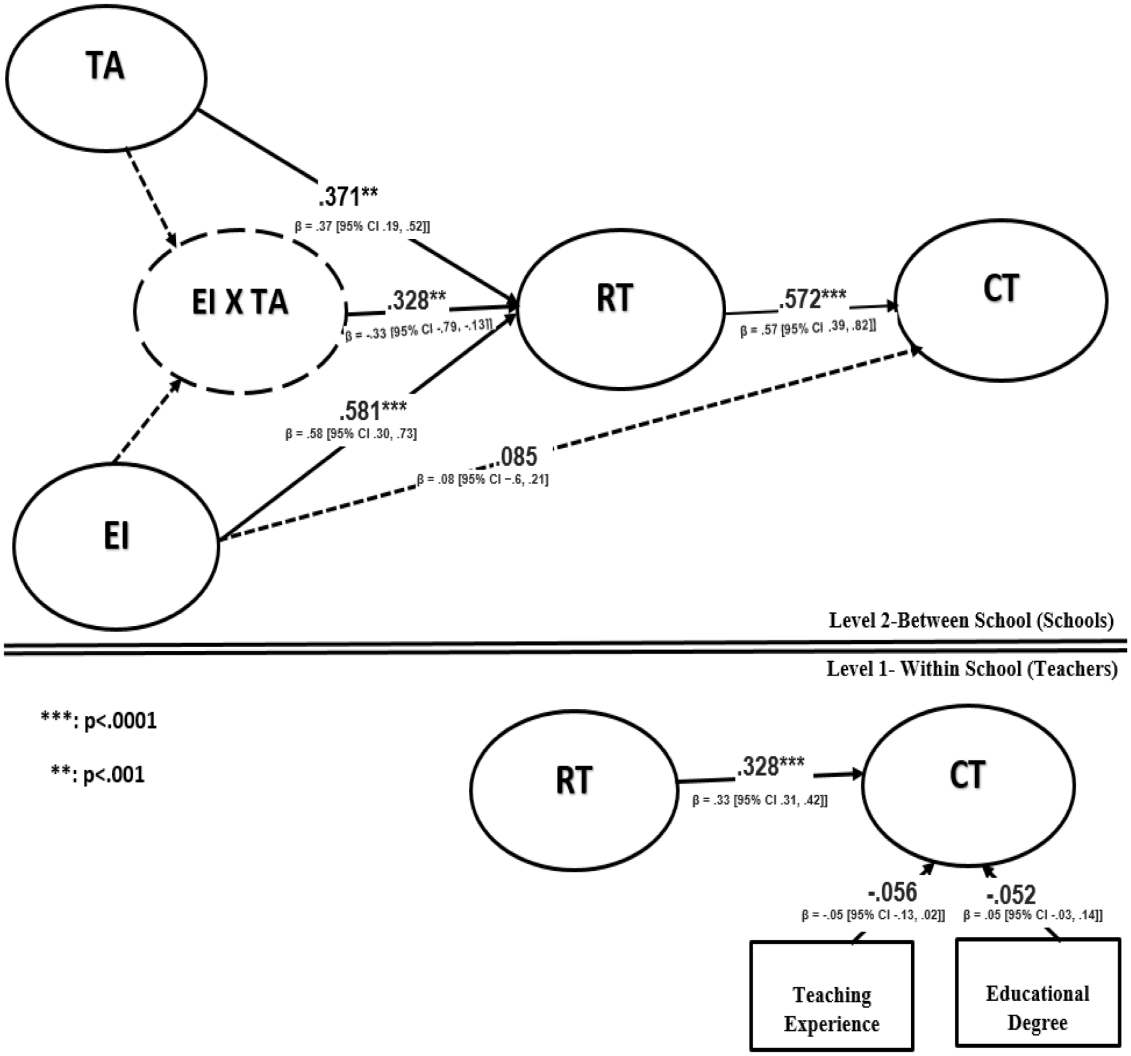
“Multilevel SEM” analysis results.

The moderating, indirect, and direct effects among our variables are listed in Figure 2.

### 2.6 Investigating the mediation model

In this phase, we examined “Hypotheses 1–4.” RT had a favorable and considerable effect on CT at both the teacher levels (b1, *γ* = 0.328, “95% CI” [0.306, 0.419], f2 = 0.41, large effect size) and school level (b2, γ = 0.572, “95% CI” [0.389, 0.823]), affirming Hypotheses 1a and 1b. EI demonstrated favourable and direct effects on RT (a1, γ = 0.581, “95% CI” [0.295, 0.727], f2 = 0.43, large effect size), supporting Hypothesis 2. The major mediating role of RT in the indirect effect of EI on CT was clear (a1b2, γ = 0.169, “95% CI” [0.085, 0.289] f2 = 0.15, medium effect size), affirming Hypothesis 4, whereas the direct effect of EI on CT was negligible (c′, γ = 0.078, “95% CI” [−0.689, 0.209], f2 = 0.08, small effect size), confuting Hypothesis 3.

### 2.7 Investigating the moderation model

Additionally, we sought to determine how TA moderated the effect of EI on RT. In support of Hypothesis 5, we discovered that the interaction between EI and CT had a substantial effect on RT (a3, γ = −0.328, 95% CI [−0.789, −0.129] f2 = 0.41, large effect size). According to a straightforward slope test, high TA scores (+ 1 SD) showed a greater association between EI and RT than low TA values (− 1 SD), supporting Hypothesis 5.

### 2.8 Investigating the moderated mediation model

In the last phase, we sought to determine whether and how TA moderated our mediation model (± 1 SD). As a result, it was clear that TA had a considerable moderating effect (a3b2, γ = 0.279, 95% CI [0.183, 0.489]) as indicated in Table 5. The indirect effect of EI on CT was only significant (γ = 0.189, 95% CI [0.088, 0.289], f2 = 0.08, small effect size) when TA was at one SD above the mean (+ 1 SD), whereas the indirect effect of EI on CT was non-significant (γ = 0.078, 95% CI [−0.032, 0.175], f2 = 0.08, small effect size) when TA was at one SD below the mean (−1 SD). As a result, our results support Hypothesis 6 by demonstrating that “the indirect effect of EI on CT is stronger when the level of TA is higher.”

**Table 5:** Conditional indirect effects of EI on CT at various levels of TA.

Moderator level	γ	SD	95% LLCI	95% ULCI
Moderated mediation index	0.279	0.073	0.183	0.489
+ 2 SD	0.235	0.068	0.129	0.387
+ 1 SD	0.189	0.049	0.088	0.289
Mean	0.124	0.043	0.054	0.228
−1 SD	0.078	0.047	−0.032	0.175
−2 SD	0.017	0.069	−0.128	0.129

LLCI, lower limit confidence interval; ULCI, upper limit confidence interval.

## 3 Discussion, implications, and conclusion

Our first hypothesis proposed that “RT will be positively related to CT.” Our data confirmed this, suggesting that RT is positively related to CT. The confirmation of the positive relationship between RT and CT aligns with and extends the contemporary understanding of teacher professional practice. RT enables educators to systematically review, evaluate and refine their pedagogical decisions, creating a cognitive metacognitive loop that fosters instructional innovation. As research suggests, when teachers engage in deep reflection on their beliefs, student responses and classroom processes, they are better positioned to design novel and adaptive teaching strategies that support creativity in the classroom (Gao et al., 2025). In this sense, RT acts not just as a compliance task but as a generative mechanism for CT—transforming experiential insights into pedagogical experimentation. Moreover, the practical linkage between reflection and creativity is substantiated in studies indicating that reflective mindsets facilitate the translation of teachers’ insights into flexible and contextually responsive instructional designs. For example, in a quasi experimental study among Chinese pre service teachers by Gao et al. (2025), the use of knowledge building and reflective journaling activities improved both teachers’ reflective orientations and their implementation of CT practices. This empirical evidence supports the notion that RT provides the cognitive scaffolding necessary for teachers to depart from routine pedagogies and engage students through invention, adaptation and problem posing tasks—all hallmarks of CT. From a professional development and policy perspective, the implication of this finding is significant. It suggests that efforts to enhance CT should not focus solely on creativity techniques or tools but also on strengthening teachers’ capacity for reflection. Professional development programmes that foster structured reflective routines, peer observation dialogues and reflective journaling can therefore serve as foundational levers for CT. This aligns with calls in the literature that reflective practice and CT should be coupled within teacher preparation and continuing education (Maor et al., 2024). Accordingly, schools and educational leaders might prioritize building reflective school cultures—where reflection becomes habitual and embedded—as a prerequisite for the systematic advancement of creative instructional practices.

The present empirical confirmation that EI will be positively related to RT aligns with and extends theoretical perspectives emphasizing the role of emotional competence in teachers’ professional practice. Teachers with higher EI are better able to perceive and regulate their own emotions and those of their students, enabling them to engage more fully in reflective processes—planning, monitoring, analysing, and adapting their pedagogy (Ferdosipour and Salimi, 2023). These emotional skills provide the affective and cognitive foundation required for RT: by recognizing emotional cues in the classroom and interpreting them thoughtfully, teachers are more likely to pause, reflect on the implications of classroom dynamics, and modify their strategies accordingly. Moreover, this relationship underlines the notion that RT is not solely a cognitive metacognitive endeavor but also an emotionally embedded practice. As the recent conceptual model of teacher emotional competence suggests, competencies such as emotional awareness, emotion regulation, and social understanding are essential for instructional and relational purposes (Savina, et al., 2025). These emotional competencies equip teachers to engage in the reflective cycle of action, observation, reflection, and modification with greater sophistication and responsiveness. Accordingly, higher EI is expected to foster more effective RT, as teachers with elevated EI are better positioned to interpret their reflections and translate them into meaningful pedagogical actions. Practically, these findings underscore significant implications for teacher education and professional development initiatives. Interventions designed to develop EI may not only improve teachers’ well being or general classroom relationships but may also pave the way for deeper RT practices. Embedding EI training within teacher preparation curricula and continuous professional development could thus boost teachers’ capacity for reflection, which in turn may lead to more adaptive, innovative instructional behaviors. Schools and educational leaders should consider supporting structures—such as reflective journals, peer observation cycles, and emotional regulation workshops—that facilitate the emotional–reflective linkage to strengthen pedagogical practice in teacher teams.

The present finding that EI did not show a straightforward positive relationship with CT challenges prevailing assumptions in the literature and invites a deeper theoretical and contextual exploration. Although a number of studies assert a direct link between EI and creative behaviors (Durnali et al., 2023), the absence of such a relationship in our data suggests that emotional competencies alone may not suffice to foster creative pedagogical practices. This may reflect the possibility that the translation of emotional awareness and regulation into CT requires additional mediating or moderating conditions—such as reflective practice, autonomy, or institutional support—that were not fully active in the current context. Several contextual factors may explain why EI did not predict CT in our sample. First, CT is a complex, multi dimensional construct that demands not just internal emotional resources, but also external structural conditions—such as professional autonomy, availability of resources, and a culture of experimentation (Gao et al., 2025). Without these enabling conditions, emotionally intelligent teachers may still revert to familiar routine practices rather than engaging in risk taking or innovation. Second, measurement issues may play a role: while EI often captures general emotional competencies, CT may depend more specifically on domain based creative self efficacy or disciplinary creativity, which were not always the focus of EI instruments used in prior work (Shi et al., 2025). Third, it is possible that EI influences CT indirectly—via intervening processes like RT or professional learning communities—which means a simple direct effect may not be observable without modeling those links explicitly (Jiang and Tong, 2025). The implications of this finding for research and practice are notable. For researchers, the result suggests that models linking EI to CT should treat CT as a distal outcome, not a direct one, and incorporate mediating or moderating variables accordingly. For practitioners and policy makers, the finding signals that professional development efforts focusing on EI alone may not guarantee increased CT. Instead, emotional training should be embedded within broader systems of reflective practice, pedagogical freedom, and innovation friendly school cultures. By doing so, schools may better harness the potential of EI to contribute to creative instructional practices.

The finding that EI influences CT indirectly through RT corroborates theoretical accounts that position reflection as the cognitive-metacognitive mechanism translating personal competencies into observable pedagogical innovation. Emotionally intelligent teachers are better able to attend to and regulate their own affective states and to interpret students’ emotional cues; these capacities facilitate deeper, more systematic reflection on instructional choices, classroom interactions, and learning outcomes (Ferdosipour and Salimi, 2023). RT, in turn, provides the procedural scaffold through which emotional insight becomes lesson redesign, differentiated questioning, and risk-taking in task design—core components of CT (Wei and Chuang, 2024). In short, EI supplies the emotional aperture for noticing and valuing classroom signals, and reflection supplies the translation machinery that converts those signals into creative pedagogical action.

Empirical and conceptual studies from recent years lend convergent support to this indirect pathway. Chain-mediation and sequential models in teacher-education research show that EI predicts intermediary constructs—such as psychological empowerment, career commitment, and reflective engagement—which subsequently promote innovative or creative instructional behaviors (Jiang and Tong, 2025). Complementary intervention research demonstrates that knowledge-building and reflective journaling raise both reflective orientations and subsequent CT practices, highlighting reflection’s mediating function between intrapersonal dispositions and enacted creativity (Gao et al., 2025). Moreover, broader investigations of creativity show that emotional competencies often relate to creativity via self-efficacy and metacognitive strategies rather than as simple direct effects, indicating that distal outcomes such as CT are frequently realized through proximal reflective processes (Durnali et al., 2023; Shi et al., 2025). Practically, these results suggest that professional development aimed at enhancing teachers’ creative instruction should adopt an integrated approach: developing emotional competencies alone may be insufficient unless programmes deliberately cultivate reflective routines and structures that enable teachers to convert emotional insight into pedagogical innovation. Interventions that combine EI training with reflective practices (e.g., guided reflective journals, structured peer observation and debrief, and facilitated lesson study) are therefore promising avenues for producing durable gains in CT (Gao et al., 2025; Wei and Chuang, 2024). Methodologically, future research should utilize longitudinal or experimental designs to investigate causal sequences and explore boundary conditions, such as teacher autonomy and school leadership, that may amplify or attenuate the pathway from EI to reflection and subsequently to CT.

The present finding—that TA strengthens the positive association between EI and RT—clarifies how personal and contextual resources jointly determine teachers’ reflective engagement. EI supplies teachers with intrapersonal and interpersonal capacities (e.g., emotional awareness, regulation, and empathic perception) that make them more likely to notice affective cues and to appraise classroom events thoughtfully (Ferdosipour and Salimi, 2023). However, our moderated result indicates that these capacities are more likely to translate into systematic reflective practice when teachers perceive sufficient professional discretion to act on those insights. This aligns with intervention and correlational studies showing that EI promotes reflective thinking and professional enquiry, but that the enactment of reflection into practice often depends on enabling institutional conditions (Aponte et al., 2025; Dogham et al., 2025). Contextualizing the moderation effect through theory helps explain its mechanism. From a self-determination and professional agency perspective, autonomy provides the decision latitude and psychological permission necessary for reflective cycles (action → observation → reflection → revision) to produce pedagogical change. When autonomy is high, emotionally intelligent teachers can experiment with lesson designs, solicit feedback, and implement the adjustments they derive from reflection; when autonomy is low, reflection may remain internalized or constrained by prescriptive curricula and accountability demands (Alshaye, 2024; Núñez-Regueiro et al., 2025). Empirical work supports this interactive logic: studies report that TA and school supports amplify the effects of dispositional resources (such as EI) on adaptive and innovative teaching behaviors, suggesting that autonomy functions as a contextual catalyst rather than a simple background correlate (Shi et al., 2025).

Practically, these results point to dual-track interventions for strengthening RT: (1) build teachers’ emotional competencies (through EI-oriented workshops, coaching, or reflective emotion-regulation exercises), and (2) reform school-level conditions to be more autonomy-supportive (e.g., flexible curriculum enactment, trust-based leadership, time for collaborative reflection). Professional development that combines EI skill development with structures that increase enactment opportunities (peer lesson study, protected planning time, decentralized curricular choices) should be especially effective because it addresses both the intrapersonal and institutional prerequisites for sustained reflection (Aponte et al., 2025; Alshaye, 2024). Future research should test these combined interventions experimentally and probe boundary conditions (e.g., subject area, career stage) that might alter the strength of the TA moderation.

The empirical confirmation of a conditional indirect effect in which EI enhances CT through RT—with this pathway becoming stronger as TA increases—contributes significantly to our understanding of how educator attributes and contextual factors combine to drive instructional innovation. From a theoretical perspective, emotionally intelligent teachers are equipped with the capacity to perceive and regulate emotions, engage empathically with students, and adapt to classroom dynamics. These emotional resources alone, however, do not guarantee innovative pedagogical behavior. RT functions as a critical mediator: it transforms emotional insights into instructional decisions, enabling teachers to systematically review their practice, iterate on lesson design, and experiment with creative strategies (Ferdosipour and Salimi, 2023; Wei and Chuang, 2024). The inclusion of TA as a moderator further highlights the context-sensitive nature of this mediated pathway: when teachers are afforded greater discretion over curriculum, pedagogy, and assessment, the relationship between EI and RT—and consequently the subsequent link from reflection to CT—is markedly strengthened. Practically, the moderated-mediation finding has three immediate implications for teacher education and school leadership. First, professional development should adopt a dual focus: enhancing teachers’ emotional competencies and strengthening their reflective capacity. EI training helps teachers notice and manage affective processes in the classroom, but without reflective mechanisms (e.g., journaling, peer discussion, lesson study), these competencies may not translate into innovation. Second, schools must foster autonomy-supportive environments: leadership practices, resource allocation, and curriculum flexibility that give teachers genuine decision-making authority enhance the likelihood that reflective thoughts will become creative instructional actions (Alshaye, 2024). Third, intervention design should explicitly address the pathway structure: for maximal impact, programmes might integrate EI training, scaffolded reflective cycles, and increased autonomy simultaneously, rather than treating these elements in isolation. This integrated approach aligns directly with the result that the CT outcome emerges only when EI is transformed via reflection and when the teacher operates in a context of high autonomy.

Finally, for research and theory, the result highlights the necessity of modeling complex, multi-stage pathways rather than relying solely on direct effects. Many earlier studies assumed that EI would directly lead to CT, but the absence of consistent direct effects in many contexts points to intervening mechanisms and boundary conditions (Gao et al., 2025; Shi et al., 2025). By demonstrating that RT mediates and autonomy moderates the EI–CT association, this work extends models of teacher innovation and professional agency, aligning with frameworks that emphasize dynamic interplay between teacher characteristics, cognitive-metacognitive processes, and institutional contexts. Future research should explore temporal dynamics (e.g., longitudinal designs) and the possibility of multi-moderator models (e.g., school culture, leadership style) to further unpack when and where EI becomes operationalized into creative pedagogy.

### 3.1 Limitations and future research

The current study has several limitations to be regarded in terms of interpretation of findings. First, our findings were limited to a sample of teachers across twenty-four cities in Türkiye which were chosen depending on the “convenience sampling” procedure. Thus, they cannot be regarded as a true representative of the population, which makes the generalizability of this study limited. Second, our data collection instruments were limited to four scales based on teachers’ opinions, which potentially makes the data less objective. Despite the fact that Dornyei (2007) states that distributing scales can supply a substantial amount of data in a brief amount of time, the acquired data is rather superficial, which limits the scope of the study. Future research can investigate the relationships among these variables using mixed approaches or qualitative approaches such as in-depth interviews and reflective journals. Furthermore, the dynamic nature of these variables may also be clarified through long-term studies on how teachers’ EI, RT, CT, and TA develop and alter during the teaching process. Teaching effectiveness cannot be increased simply by giving teachers a wealth of technical and pedagogical knowledge. The development of psychological traits including EI, reflectivity, creativity, and autonomy must be encouraged. Thus, teachers should take into account these psychological and cognitive aspects of their professional traits in their teaching.

## Data Availability

The raw data supporting the conclusions of this article will be made available by the authors, without undue reservation.
