# Correction: Understanding how emotional intelligence shapes teachers' reflective and creative teaching: the moderating role of teacher autonomy

**DOI:** 10.3389/fpsyg.2026.1810128

**Published:** 2026-02-23

**Authors:** Şenol Orakcı, Osman Aktan, Hüseyin Çevik

**Affiliations:** 1 Faculty of Education, Curriculum and Instruction, Aksaray University, Aksaray, Türkiye; 2 Faculty of Education, Special Education, Düzce University, Düzce, Türkiye; 3 Faculty of Education, Curriculum and Instruction, Zonguldak Bülent Ecevit University, Zonguldak, Türkiye

**Keywords:** creative teaching, emotional intelligence, reflective teaching, teacher autonomy, teachers

Affiliation 2. Faculty of Education, Special Education, Düzce University, Düzce, Türkiye was erroneously given as 2. Faculty of Education, Curriculum and Instruction, Düzce University, Düzce, Türkiye.

The original version of this article has been updated.

